# Pediatric flecainide pharmacogenomics: a roadmap to delivering precision-based care to pediatrics arrhythmias

**DOI:** 10.3389/fphar.2024.1477485

**Published:** 2024-12-16

**Authors:** Ronald Palmen, Mollie Walton, Jonathan Wagner

**Affiliations:** ^1^ Department of Pediatrics, University of Missouri-Kansas City School of Medicine, Kansas City, MO, United States; ^2^ Division of Cardiology, Kansas City, MO, United States; ^3^ Division of Clinical Pharmacology, Toxicology and Therapeutic Innovation, Children’s Mercy, Kansas City, MO, United States

**Keywords:** anti-arrhythmics, flecainide, ontogeny, pediatrics, pharmacogenomics, supraventricular tachycardia

## Abstract

Flecainide acetate is a Class 1c anti-arrhythmic with a potent sodium voltage gated channel blockade which is utilized for the second-line treatment of tachyarrhythmias in children and adults. Given its narrow therapeutic index, the individualization of drug therapy is of utmost importance for clinicians. Despite efforts to improve anti-arrhythmic drug therapy, there remain knowledge gaps regarding the impact of variation in the genes relevant to flecainide’s disposition and response. This variability is compounded in developing children whose drug disposition and response pathways may remain immature. The purpose of this comprehensive review is to outline flecainide’s disposition and response pathways while simultaneously highlighting opportunities for prospective investigation in the pediatric population.

## Introduction

The last three decades have brought extensive reforms in pediatric drug labeling due to changes in US regulations and new federal laws (Food and Drug Administration, 1994; Food and Drug Modernization Act, 1997; Steinbrook, 2002). The Food and Drug Administration (FDA) has issued over 1000 pediatric study requests since 1998 (Green et al., 2021). Despite early success with pediatric study requests resulting in new or expanded pediatric labeling (Wharton et al., 2014), gaps in pediatric cardiovascular drug labeling remain (Pasquali et al., 2008). Even with the success of this legislation in obtaining pediatric labeling for numerous pharmacotherapeutics, the gaps reveal the complexity of pediatric drug dosing, efficacy, and toxicity (Rodriguez et al., 2008).

Clinicians of pediatric medicine uniformly appreciate austerity in the appropriate diagnosing and treatment of growing and developing children. The untrained observer may merely accept adult treatment guidelines extrapolated to a younger population. However, such linear extrapolation ignores the influence of development on the expression of proteins responsible for drug disposition and, by extension, the impact that has on the dosing requirements and safety profiles of drugs utilized from birth until young adulthood. The impact of ontogeny on drug disposition has been accelerated by *in vitro* and *in vivo* data generated on the developmental pattern of drug-metabolizing enzymes (Hines, 2008; Hines, 2007; Hines and McCarver, 2002; McCarver and Hines, 2002) and transporters (Prasad et al., 2016). Even with a full understanding of the ontogenic trajectory of a certain drug’s disposition pathway, an understanding of genetic variation on drug disposition and efficacy in the developing child is lacking (Leeder and Kearns, 2002; Leeder, 2003; Leeder and Kearns, 2012; McLaughlin et al., 2019). The limited number of participants and decreased involvement of a vulnerable population has halted further prospective pediatric trials (Ward and Sherwin, 2015; Stiers and Ward, 2014) and, thereby, the understanding and incorporation of pharmacogenomic principles into clinical practice.

In the absence of more comprehensive data, a systemic approach has been developed to gather more information about certain drugs and identify knowledge gaps to strategize the design of future studies. This approach has previously been utilized to address the dilemma of over-the-counter cough and cold preparations (Leeder et al., 2010), 3-hydroxy-3-methyl-glutaryl coenzyme A reductase inhibitors (statins) (Wagner and Leeder, 2012), and more recently, beta blockers (Walton and Wagner, 2024). Ultimately, this review will take the reader through a systematic approach that reveals the current knowledge regarding the impact of ontogeny and genetic variation on the dose–exposure–response continuum of a drug with a narrow therapeutic index.

## Treatment of childhood tachyarrhythmias

Supraventricular tachycardia (SVT) is the most common childhood tachyarrhythmia (Weindling et al., 1996) and the most common tachyarrhythmias requiring treatment in pediatrics (Garson et al., 1981). It is a common pediatric condition with an incidence estimated to be 1 in 250–1000 children (Weindling et al., 1996). Our knowledge base regarding the mechanism and diagnosis of childhood tachyarrhythmias has improved dramatically over the last 30–40 years (Vignati, 2007). Despite its relative common occurrence in pediatrics, there remains insufficient data to guide treatment, thus highlighting prescription inconsistencies in drugs used in pediatrics (Wong et al., 2006; Seslar et al., 2013). This knowledge gap is even more pronounced in special populations (e.g., neonatal) where rapid growth and development may influence the diagnosis and treatment of clinical disease (Kearns et al., 2003). With advances in catheter-based options for treating tachyarrhythmias in older children, the demand for precision-based pharmacotherapy is thus more prominent in the younger pediatric population where rapid growth and development occur.

Current management strategies are based on observational data and clinician experience (Weindling et al., 1996; Wong et al., 2006; Seslar et al., 2013) rather than randomized, prospective trials or systematic reviews. Unfortunately, dosing guidelines for many of these anti-arrhythmics are extrapolated from adult recommendations, similar to many other pediatric cardiovascular drug agents (Pasquali et al., 2008; Maltz et al., 2013). This allometric approach (i.e., based on relative body size) assumes that human growth is a linear process. However, age-associated changes in body composition, organ function, and the pharmacologic mediators of drug disposition/response must be taken into consideration to individualize the dose needed for desired exposure to maximize efficacy and minimized adverse events. The variability of drug exposure places the patient at risk of treatment failure or adverse events, thereby prolonging hospitalization and the utilization of intensive care services.

The majority of infants with incessant arrhythmias clinically respond (i.e., absence of breakthrough tachycardia) to first line agents such as propranolol and digoxin (Weindling et al., 1996; Van der Merwe and Van der Merwe, 2004). However, 40%–45% of infants have breakthrough tachycardia that requires additional therapy (Seslar et al., 2013). The most common second-line agents for the chronic management of SVT (e.g., amiodarone, flecainide, and sotalol) have demonstrated efficacy in controlling tachycardia, but their narrow therapeutic index requires careful dose titration and close observation, lengthening hospitalization in acute care settings. In fact, adding a second-line agent can extend the length of stay two-fold or more (Seslar et al., 2013). Conversely, the concern of toxicity may also drive the prescribing clinician to inadequately underdose patients. In an era of fiscal strain on our healthcare system, optimized pharmacotherapy tailored to each patient, including demographic and genetic data, can not only efficiently provide more accurate dosing but lessen the resource and economic burden in acute care settings.

### Flecainide therapy in children

**FIGURE 1:**
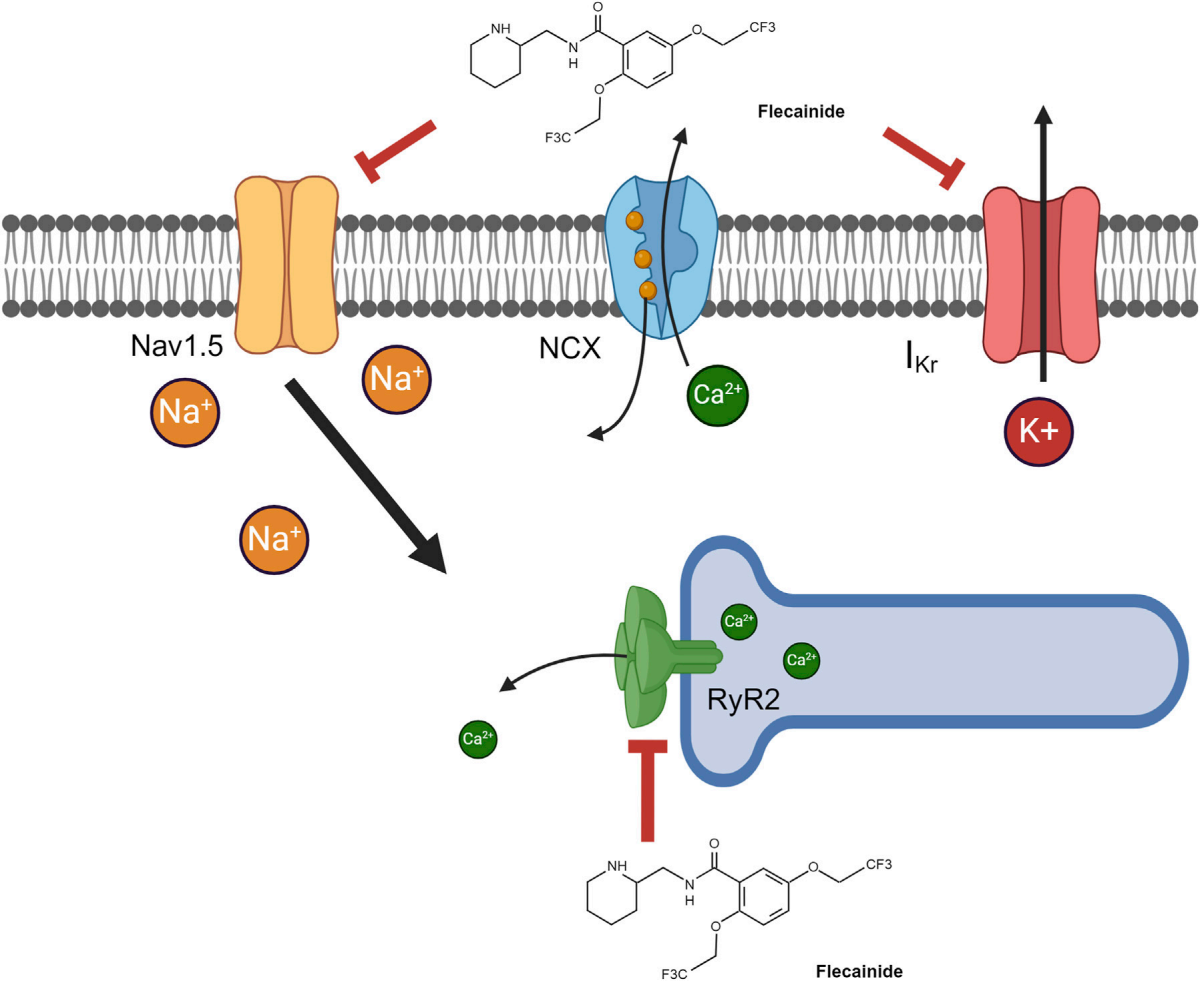
Flecainide pharmacodynamic pathways. Flecainide can antagonize three major pathways, resulting in its pharmacologic effects. The majority of its pharmacodynamic effect is through antagonism of voltage-gated sodium channels (Na_v_1.5). Alternative pathways include antagonism of 1) the ryanodine receptor (RyR2) on the sarcoplasmic reticulum, resulting in lower intracellular calcium, and 2) the delayed rectifying potassium channel (I_Kr_), resulting in diminished potassium export. Created in BioRender. Walton and Wagner (2024) https://BioRender.com/p71p206.

Flecainide acetate is a benzamide derivative (N-2-piperidnylmethyl)-2,5-bis (2,2,2-trifluoroethoxymonacetate)), class 1c Vaughn Williams anti-arrhythmic with potent sodium voltage gated channel blockade used to treat tachyarrhythmias in children and adults (Till et al., 1989; Zeigler et al., 1988; Flecainide versus quinidine for treatment of chronic ventricular arrhythmias, 1983; Wren and Campbell, 1987; Berns et al., 1987; Anderson et al., 1981; Perry and Garson, 1992; Perry et al., 1989; Aliot et al., 2011). Flecainide acetate limits the flow of sodium into the myocyte, prolonging the initial phase of action potential (Figure 1). The electrophysiological result is slowed atrial and ventricular conduction and a lengthened ventricular refractory period at low plasma levels (Hodess et al., 1979; Seipel et al., 1981), pharmacodynamically resulting in suppression of tachyarrhythmias. At high plasma levels, depolarization delay through the cardiac conduction system occurs, causing a prolonged refractory period in the myocardium which increases the risk of pro-arrhythmic events (Hodess et al., 1979; Salerno et al., 1986). This slow dissociation from the sodium channel accounts for a majority of its longer lasting therapeutic effects (Ikeda et al., 1985). Flecainide also has binding affinity—albeit lower than sodium channels—to the delayed rectifying potassium channel (I_Kr_) (Follmer and Colatsky, 1990; Follmer et al., 1992) and cardiac ryanodine receptor 2 (RyR2) to block their respective ion movement (Ikeda et al., 1985; Salvage et al., 2022; Kryshtal et al., 2021) (Figure 1). In patients with catecholaminergic polymorphic ventricular tachycardia (CPVT) where there is enhanced cardiac RyR2 activity, excessive calcium efflux from the sarcoplasmic reticulum increases the risk of ventricular arrhythmias (Priori et al., 2002). Flecainide is known to provide excellent treatment for preventing ventricular arrhythmias in CPVT (Watanabe et al., 2009; Watanabe et al., 2013; Kannankeril et al., 2017); it was recently demonstrated mechanistically that flecainide directly antagonizes RyR2, leading to its effectiveness in CPVT (Kryshtal et al., 2021).

Flecainide acetate was initially developed in 1972 as a fluorinated local anesthetic analog of procainamide (class 1a antiarrhythmic) (Hudak et al., 1984). However, further evaluation in animal models discovered its anti-arrhythmic properties (Kvam et al., 1984). Clinical testing and development for human use occurred in the late 1970s by Riker Laboratories (3M) and it was marketed under the trade name (Tambocor). It came off patent in 2004 and is now available in several generic forms. Despite its use for over 30 years, there is still inadequate pediatric labeling for this agent despite its common use for refractory tachyarrhythmias (Wong et al., 2006; Seslar et al., 2013). It is available in intravenous and oral dosage forms, but it is predominantly given orally in tablet or solution. The use of flecainide has been limited in some settings, particularly in patients with acute heart failure and cardiomyopathy due to an observed increase in mortality after its use in post-myocardial infarction patients as a part of the multi-center Cardiac Arrhythmia Suppression Trial (CAST) (Echt et al., 1991). The result of this study has been extrapolated to pediatric congenital heart disease (CHD), resulting in decreased use in the CHD population since the 1990s. However, recent analysis showed an increased trend in flecainide use in this pediatric CHD population over the last decade (Moffett et al., 2015).

Most pediatric trials have focused on the efficacy of arrhythmia suppression and safety (Perry and Garson, 1992; Fish et al., 1991; Till et al., 1987). In the largest analysis by Perry and Garson (1992), efficacy, defined by acute arrhythmia suppression, reduction of arrhythmia frequency, and reduction of incessant tachycardia, was achieved with flecainide in 70%–80% of patients. However, 20%–30% failed to respond to it. Plasma flecainide levels with an adequate control of arrhythmias had a range of 200–500 ng/mL (Perry and Garson, 1992). It is unknown if those patients with failed efficacy achieved the targeted plasma exposure necessary for success. Till et al. (1989) observed a large degree of variability of dosing in weight-based and dosing by body surface area, with poor correlation between the dose received to exposure obtained among infants and children on chronic flecainide therapy. Flecainide has also been used in the treatment of fetal supraventricular tachycardia (Tamirisa et al., 2022; Miyoshi et al., 2019; Strizek et al., 2016; Ekiz et al., 2018; Allan et al., 1991) with very little peripartum flecainide toxicity reported due to wider therapeutic drug monitoring in the management of fetal arrhythmias (Hall and Ward, 2003). Interindividual variation has been observed in adults (Homma et al., 2005) due to additional co-morbidities (e.g., liver and/or renal impairment) (Forland et al., 1988; McQuinn et al., 1988) or other factors that disrupt flecainide disposition (e.g., ADME: absorption, distribution, metabolism, excretion) (Caplin et al., 1985; Padrini et al., 1993; Zordan et al., 1993; Doki et al., 2006; Doki et al., 2009; Lim et al., 2008; Lim et al., 2010). It is very plausible that other factors—ontogeny and genetic variation—contribute to the variability observed in flecainide disposition and response in developing children. Those specific factors are discussed in more detail below.

### Impact of ontogeny and genetic variation on flecainide disposition and response

There is a paucity of evidence regarding pediatric drug disposition, which limits optimal pediatric drug-dosing strategies to best effect and least potential toxicities (i.e., dose-optimization). Given the continued number of pediatric tachyarrhythmia cases per year, the pediatric cardiology community needs to develop dose optimization guidelines tailored to the individual pediatric patient. To achieve this goal, a full understanding of the dose–exposure–response continuum in pediatric patients dosed with flecainide must be understood, and subsequent studies are needed to address knowledge gaps and leverage existing data related to ontogeny and the genetic variation of relevant drug disposition pathways. The purpose of the remainder of this review is to present three topics that should be considered when assimilating current knowledge for application to problems related to variability in drug disposition and response in children. Such a systematic approach has been previously used to address the knowledge gaps for other pediatric therapeutics agents (Leeder et al., 2010; Wagner and Leeder, 2012; Walton and Wagner, 2024) and will be applied here to identify the knowledge deficits related to the contribution of ontogeny and genetic variation on flecainide disposition and response in children, with implications for the design of prospective clinical trials.

## Essential concepts for evaluating variability in flecainide disposition and response in children

1. Existing data regarding drug properties and gene products that are quantitatively relevant in the disposition (absorption, distribution, metabolism, and excretion) and response of flecainide therapy.

Essential to this systematic review is knowledge of the necessary patient and drug properties that could influence drug disposition. For example, flecainide is a highly lipophilic drug agent (logP 3.8) and should hypothetically demonstrate less dependency on protein-mediated cellular translocation (e.g., drug transporter influx/efflux); it is thus more likely to passively diffuse across the cellular lipid bilayer for distribution (De Schryver et al., 2019). Flecainide should have a lower systemic exposure (e.g., plasma drug concentration) and larger volume of distribution than other anti-arrhythmic drugs that are more hydrophilic (e.g., atenolol) (Kirch and Gorg, 1982).

Flecainide easily distributes into plasma with modest protein binding (approximately 35%–55%) (Zordan et al., 1993; Conard and Ober, 1984). There is an equivocal amount of literature to suggest whether flecainide, as a basic drug, has preferential binding to albumin *versus* α1-acid glycoprotein (Zordan et al., 1993; Conard et al., 1984). In healthy adults, there is very little inter-individual variability (age or gender) in regards to protein binding (Zordan et al., 1993). Conversely, in a developing infant, the level of serum proteins is reduced relative to adults (Wagner and Abdel-Rahman, 2013; Cartlidge and Rutter, 1986; Kanakoudi et al., 1995). Overall, the relative degree of free flecainide could be higher and could thereby influence the dose–exposure–response relationship. Protein binding can be altered in adults after myocardial infarctions, with more α1-acid glycoprotein available for protein binding. However, flecainide is a more poorly bound basic compound, so there is 20% increase in free drug concentration after acute myocardial infarction (Caplin et al., 1985). Perhaps this was the factor that contributed to increased mortality in the CAST trial. In CHD, it is unclear whether the concentration of serum proteins is decreased even further than neonates with structurally normal hearts. The degree of protein binding and lipophilicity should be considered for optimizing drug treatment; however, there is no data to suggest that a specific gene is responsible for dose-exposure in this respect. Finally, there has been a high correlation (∼80–100%) of maternal and fetal plasma flecainide concentrations late in pregnancy (i.e., third trimester) (Allan et al., 1991; Bourget et al., 1994), suggesting a robust transplacental transport of flecainide to the fetus.

An additional drug-specific factor is the stereoselectivity of flecainide, which is a racemic mixture of R (−) and S (+) enantiomers (Gross et al., 1989) with equivalent ventricular antiarrhythmic properties in animal models (Banitt et al., 1986). In animal cardiac myocyte models, there is a greater decrease in the maximum upstroke velocity of the action potential in Purkinje fibers with the S (+) enantiomer than the R (−) but no difference in action potential amplitude or conduction time (Smallwood et al., 1989). However, there are still inadequate data to support stereoselectivity influencing response in humans. Additionally, these data support only sodium channel antagonism and do not evaluate the RyR2 and I_Kr_ effects. These alternative flecainide receptors must be explored in future research. The relevant patient-specific (i.e., flecainide disposition) pathways are described in detail below and summarized in Table 1.

**TABLE 1:** Flecainide drug distribution pathways.

	Absorption	Distribution Hepatic/renal uptake	Metabolism Phase 1	Metabolism Phase 2	Excretion Efflux
Flecainide	• Passive diffusion (major)	• Passive diffusion	• CYP2D6, R(−)>S(+)!	• Unknown	• Passive diffusion
	• H+ tertiary amine antiporter*• MDR1 (P-gp)*• OCT2*• PEPT1*	• OCT2 (kidney)*	• CYP1A2, S(+)>R(−)#		• MDR1 (P-gp)*

*unknown impact; ^!^major pathway, ^#^minor pathway.

### Absorption

Given its lipophilic nature, flecainide displays near complete absorption in its unchanged form from the gastrointestinal tract (Conard and Ober, 1984) and does not undergo any consequential pre-systemic biotransformation, with greater than 90%–95% of the unchanged dose reaching systemic circulation (Conard and Ober, 1984). The time to peak concentration (t_max_) is 2.7±1.5 h in children and is similar in adults (Perry et al., 1989; Conard and Ober, 1984). However, Till et al. (1989) reported a 1-month-old infant with a tmax of 4.8 h and maximal concentration very near toxicity, showing that variability in the neonatal population does exist. Flecainide toxicity has previously been reported in the neonatal period, and this could be a constellation of developmental patient-specific factors (e.g., protein binding, absorption rates, peripheral tissue distribution, differential metabolism) (Palmen et al., 2023; Karmegaraj et al., 2017; Jang et al., 2013; Poh et al., 2020; Romain et al., 1999; Ackland et al., 2003). This degree of variability regarding absorption in the neonatal and younger infant population as referenced by Till et al. (1989) does require further investigation. The prompt gastrointestinal absorption of the majority of patients suggests that the mechanism could also occur via a transporter-mediated process; however, there are few *in vitro* and *in vivo* data to suggest a definitive mechanism of absorption across the enterocyte. It is notable that the absorption of flecainide can be impaired with the co-ingestion of milk (Russell and Martin, 1989; Thompson et al., 2012). The human peptide transporter (PEPT) 1, encoded by *SLC15A1*, is a proton-coupled peptide cotransporter on the luminal surface of the enterocyte (Liang et al., 1995), and peptide by-products of milk proteins can inhibit this transporter (Fujisawa et al., 2006). Nevertheless, it remains unknown if flecainide is a substrate for PEPT1. Another uptake transporter, H+/tertiary amine antiporter, also appears to be involved in flecainide uptake *in vitro* (Horie et al., 2014). The excellent bioavailability profile suggests that flecainide fails to be a significant substrate for enterocyte efflux transporters contributing to pre-systemic clearance back into the intestinal lumen. Despite being a substrate and inhibitor of the organic cation transporter (OCT) 2 (Zolk et al., 2009), which is expressed on the basolateral membrane of enterocytes (Jonker and Schinkel, 2004), there are no data that suggest that this transporter affects bioavailability (Cascorbi, 2011; Nies et al., 2011; Keppler, 2011). Digoxin is a substrate for the multidrug resistance protein 1 (MDR1), alternatively known as p-glycoprotein (P-gp)) (Cascorbi, 2011), and no significant changes in digoxin pharmacokinetics have been reported when co-administered with flecainide. However, Horie et al. (2014) suggest otherwise, concluding that MDR1 may be a barrier to flecainide absorption. Absorption kinetics have been shown to affect flecainide exposure (Cmax and AUC) in solution *versus* tablet form (Deneer et al., 2004). Therefore, the rate and degree of flecainide transport across the enterocyte cellular membrane can affect systemic exposure. Flecainide’s specific mechanism of absorption and relative contribution to the dose–exposure–response continuum needs further elucidation in order to be considered a source of potential variability.

### Distribution

Drug-specific parameters related to drug distribution (e.g., lipophilicity, protein binding affinity) were described above. Xenobiotic transporters, as described earlier, can contribute to the dose–exposure–response continuum. Transporters observed in the enterocyte may also contribute to drug distribution in the hepatocyte and myocyte (Nigam, 2015; Ho and Kim, 2005). Recent studies have identified mRNA expression of at least 14 influx and efflux transporters in the cardiac myocyte (McBride et al., 2009; Grube et al., 2006). Currently, there is no compelling data to suggest that flecainide is a substrate for any of these transporters, but this requires further research. Given its lipophilic nature, most of flecainide’s hepatic uptake is associated with passive diffusion. However, given flecainide’s association with OCT2, which is primarily expressed on the basolateral cellular surface of renal tubules (Choi and Song, 2008), this protein could contribute to the observed variability in drug clearance and, by extension, systemic exposure the pediatric population. For instance, inhibition studies with cimetidine, a potent OCT2 inhibitor, have demonstrated reduced renal excretion of flecainide (Somogyi and Muirhead, 1987), and Zolk et al. (2009) describes flecainide having at least modest inhibition of OCT2. Together, these data suggest that flecainide has some degree of binding affinity to OCT2. Conversely, Horie et al. (2014) described OCT proteins not contributing to the renal transport of flecainide to renal tubule cells for elimination. Notably, there were no specific proteins (e.g., OCT1 vs. OCT2) that were illuminated in this work, and thus it cannot be definitively concluded that OCT2 fails to transport flecainide. The binding affinity and uptake of flecainide to OCT2 must be explored with future *in vitro* cellular investigation.

### Metabolism

**FIGURE 2:**
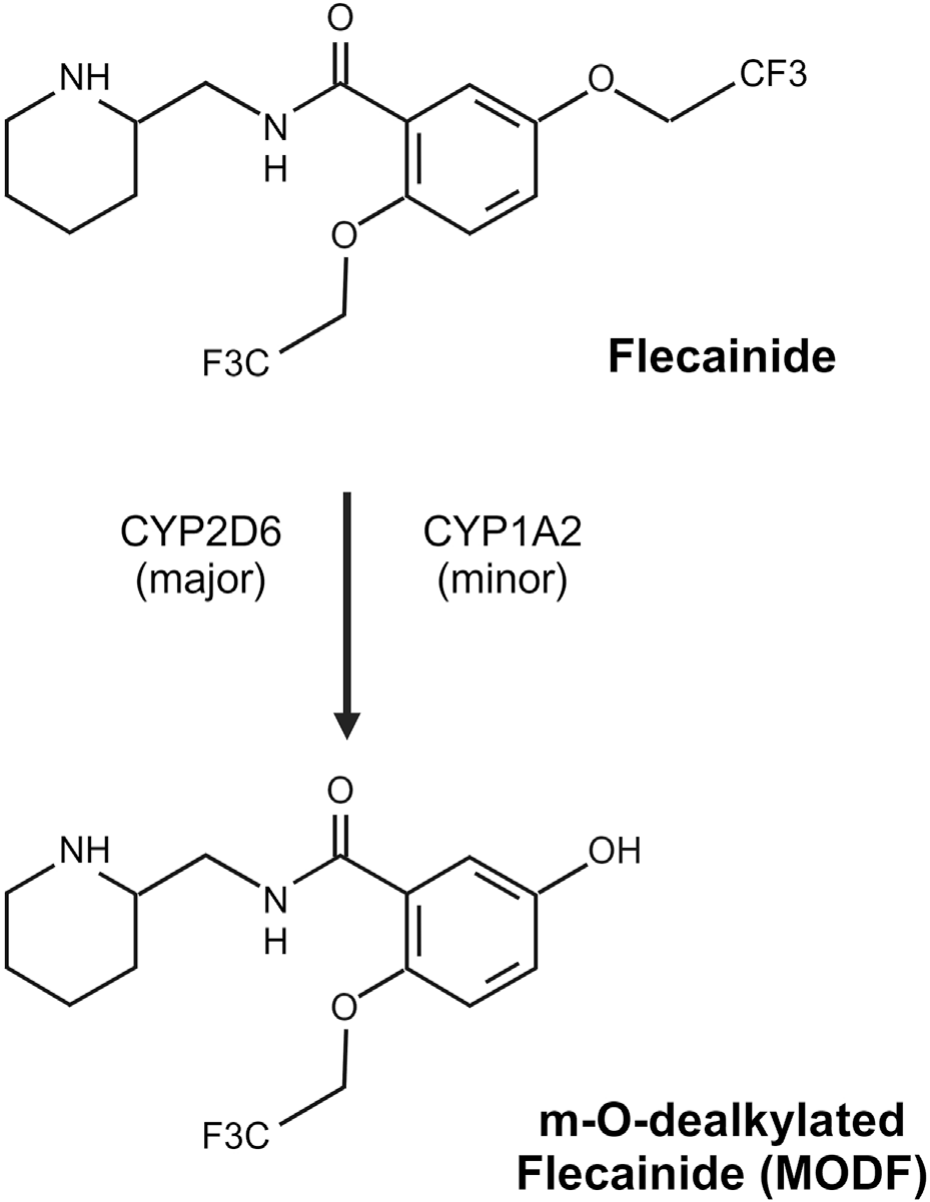
Flecainide metabolism pathway. Flecainide undergoes predominantly CYP2D6-mediated metabolism with a minor contribution from CYP1A2 to form an inactive metabolite, m-O-dealkylated flecainide (MODF). Created in BioRender. Walton and Wagner (2024) https://BioRender.com/h94f985.

Flecainide is metabolized in the hepatocyte to m-O-dealkylated flecainide (MODF) and subsequently oxidized to m-O-dealkylated lactam flecainide (MODLF) (Figure 2). These two major metabolites have low potency, with MODF having only 20% relative anti-arrhythmic activity, and MODLF has no detectable antiarrhythmic activity in animal models (Conard and Ober, 1984; McQuinn et al., 1984). Metabolism is incomplete with nearly 25%–40% flecainide acetate excreted in its unchanged form (Conard and Ober, 1984). Approximately 15% of the administered dose is recovered in the urine for each major metabolite (Conard and Ober, 1984; McQuinn et al., 1984). The elimination half-life is highly variable in adults ranging 7–30 h (Conard and Ober, 1984; McQuinn et al., 1984) and can be prolonged in different disease states (i.e., congestive heart failure, hepatic or renal impairment) (Conard and Ober, 1984). *In vitro*, CYP2D6 appears to be the most important enzyme contributing to the dealkylation of flecainide (Doki et al., 2009). CYP1A2 also contributes to MODF formation *in vitro* but to only 1/6 of that in CYP2D6 (Doki et al., 2009). *In vivo*, the conversion of flecainide to MODF via CYP2D6 has been verified, with poor metabolizers (PM) of CYP2D6 having a significantly higher area under the curve (AUC) than extensive metabolizers (EM) after a single dose (Mikus et al., 1989). CYP2D6 phenotype at steady state yielded a similar pharmacokinetic profile but unchanged pharmacodynamic parameters (e.g., QRS duration) (Funck-Brentano et al., 1994; Tenneze et al., 2002). Additionally, the co-administration of cimetidine, a potent CYP2D6 inhibitor, resulted in a ∼30% increase in flecainide AUC (Tjandra-Maga et al., 1986a), suggesting that CYP2D6 has a significant role in flecainide metabolism. However, CYP1A2 may serve as an alternative route of metabolism in CYP2D6 PMs and could mitigate the effects of *CYP2D6* genetic variation. Notably, these trials involved a small number of CYP2D6 PMs. In the larger cohort by Doki et al. (2006), the *CYP2D6* genotype did have an impact on flecainide clearance in adults with SVT. Beyond genetic variation, differences in flecainide clearance have been observed between genders, with male participants having ∼30%–35% lower systemic exposure of flecainide relative to female participants (Doki et al., 2007). This might be partially related to higher expression of CYP1A2 in male participants than female participants (Ou-Yang et al., 2000); however, sex-related differences in CYP2D6 are debated, with data to suggest no difference in some substrates (Bebia et al., 2004) and a difference noted for others (e.g., metoprolol) (Luzier et al., 1999). Stereoselective metabolism has been observed in human liver microsomes with enhanced CYP2D6 clearance (∼2.5-fold) noted for R (+) flecainide compared to S (−) flecainide (Doki et al., 2015). Additionally, there is slightly more S (−) flecainide clearance than R (+) flecainide in similar microsomal incubations with CYP1A2 (Doki et al., 2015).

The MODF and MODLF metabolites are subject to conjugation and found predominantly in their conjugated form; phase 2 metabolism is thus likely to occur with these drug substrates (Conard and Ober, 1984; McQuinn et al., 1984). However, the specific phase II metabolism enzyme (e.g., UGT, SULT) that leads to the conjugation of flecainide is unknown but requires further elucidation, as it could be a source of interindividual variability in drug biotransformation amongst patients.

### Excretion

A majority of flecainide following oral dosing is excreted in the urine as flecainide and metabolites (∼85%) and 5% in the feces, suggestive of minimal biliary excretion (Conard and Ober, 1984; McQuinn et al., 1984). Horie et al. (2014) suggest that that MDR1 may have a more important role in the renal tubular secretion of flecainide. In their cellular models, co-incubation with potent MDR1 inhibitors (quinidine, carvedilol, and amiodarone) resulted in the enhanced apical to basolateral (i.e., drug influx) transport of flecainide due to the inhibition of MDR1, which is typically expressed at the apical surface and is responsible for efflux back into the intestinal lumen or renal tubules in humans. However, it does not appear to be influential in excretion to the intestinal lumen, as the reported bioavailability is 60%–85% (Tjandra-Maga et al., 1986b); its influence on the renal excretion of flecainide remains unknown. As is the case with many other drugs, more data are needed on the contribution of drug transporters to drug disposition regarding hepatic/renal uptake and enterocyte/renal excretion.

## Response

Flecainide, as a Class Ic antiarrhythmic, predominantly inhibits cardiac voltage-gated sodium Na_v_1.5 channels which are expressed in cardiac myocytes (Fozzard and Hanck, 1996; Dong et al., 2020). This sodium channel subtype carries a unique glycosyl moiety and is composed of an α-subunit and interacts with β-subunits (Jiang et al., 2020). The pore-forming α-subunit is composed of four domains, each with six membrane-spanning segments (Catterall, 2000), and flecainide binds directly to this pore region (Jiang et al., 2020). Na_v_1.5 channels are additionally unique in that they have a loss of disulfide-bonding capability at the Na_v_β subunit-interaction sites (Jiang et al., 2020). Na_v_1.5 channels are responsible for the influx of sodium during rapid depolarization, controlling the rate and homogeneity of cardiac myocyte action potential. Flecainide is able to bind to the central cavity, partially closing the influx of sodium ions and thereby slowing cardiac conduction rates (Jiang et al., 2020). Although other sodium channels are expressed in human cardiac myocytes, if the function of Na_v_1.5 channels is diminished, the other channels cannot surmount efficient offset to prevent lethal arrhythmias (Jiang et al., 2020). Thus, Na_v_1.5 channels represent the most important channel for action-potential depolarizations. Not surprisingly, aberrations of the Na_v_1.5 protein are associated with electrophysiologic pathologies that include long QT syndrome (LQT) type 3, Brugada syndrome, congenital AV block, drug-induced LQT, atrial fibrillation, and sick sinus syndrome (Abriel, 2010). Additionally, regulatory protein (e.g., β subunit, caveolin-3, α-1-syntrophin) aberrations can result in LQT 9, 10, 12, Brugada syndrome, cardiac conduction disease, and atrial fibrillation (Abriel, 2010).

As well as slowing sodium influx resulting in slowing conduction rates, flecainide has binding affinity to the ryanodine receptor (RyR2) found on the sarcoplasmic reticulum of cardiac myocytes (Watanabe et al., 2009). RyR2 is a tetramer of four 565,000-D RyR polypeptides, each of which binds one 12,000-D FK506 binding protein (FKBP12 and FKBP12.6) as well as large cytoplasmic scaffolding amenable to channel modulation (Marks, 1996). The main mechanism of ion channel manipulation occurs through phosphorylation via protein kinase A, allowing the dissociation of FKBP12.6 from RyR2 and opening the channel access for calcium entrance to the cytosol; this is further supported by the hyperphosphorylation of RyR2 in channels which are deemed “leaky” (Marks, 1996). Flecainide’s affinity to RyR2 antagonizes calcium release into the cytoplasm—essential for excitation–contraction coupling (Watanabe et al., 2009). This pleotropic effect is particularly advantageous for catecholaminergic polymorphic ventricular tachycardia (CPVT), where a gain-of-function mutation of the RyR channels results in increased calcium influx and increasing cell excitability (Kryshtal et al., 2021). As such, flecainide, through its antagonism of RyR2, can result in diminished intracellular calcium (Kryshtal et al., 2021). However, the specific mechanism by which this reduces ventricular arrhythmias in CPVT is uncertain. The data are equivocal on whether flecainide disrupts sodium channel function in isolation (Liu et al., 2011; Bannister et al., 2016; Bannister et al., 2015; Sikkel et al., 2013) and an alternative mechanism of disruption of calcium release through the direct engagement of RyR2 (Kryshtal et al., 2021). Notwithstanding, there is a complex role for flecainide engagement with RyR2 channels. For example, there are multiple binding sites, four known inhibitory sites, and one activating site, as well as dynamic voltage dependance for each (Salvage et al., 2022). This differs from the single binding site found on NaV1 channels. Flecainide can engage with the activating site of RyR2, which may explain the former’s documented proarrhythmic effects (Salvage et al., 2021). Collectively, the specific mechanisms by which flecainide attenuates ventricular arrhythmias in patients with CPVT must be explored in order to improve individualized treatment plans and mechanisms for new drug discovery.

Although the antiarrhythmic efficacy of flecainide has primarily been attributed to its effects on the fast inward Na + current, the blockade of the delayed rectifier K+ channel (I_Kr_) channel may be complimentary to the electrophysiological effects on cardiac myocytes. At low doses, flecainide inhibits the rapid component of I_Kr_ (Follmer and Colatsky, 1990; Anno and Hondeghem, 1990; Tamargo et al., 2004). Follmer and Colatsky (1990) investigated the blockage of the I_Kr_ channel by flecainide in cat ventricular myocytes, showing that the estimated amount of I_Kr_ channel-block during the normal course of flecainide therapy could approach ∼25%–63%. The I_Kr_ channel has been identified in human atrial and ventricular myocytes but is differentially expressed with higher levels in the left atrium and ventricular endocardium (Grant, 2009). The I_Kr_ channel is characterized by rapid activation and inactivation and strong inward rectification at positive potentials (Tamargo et al., 2004). This channel plays an important role in governing the cardiac action-potential duration and refractoriness. Inhibition of the I_Kr_ channel underlies an increase in action-potential duration produced by flecainide. As such, the clinical significance of I_Kr_ blockade may relate to the former’s ability to prolong the duration of premature electrical responses elicited early in diastole (Nakaya et al., 1989; Olsson and Edvardsson, 1981). For example, Somberg et al. (1987) showed that the suppression of sustained ventricular tachycardia during electrophysiological testing has been associated with a significantly greater prolongation of refractoriness in flecainide responders than with non-responders. The I_Kr_ channel is encoded by the genes KCNH2 (α subunit; associated protein hERG) and KCNE2 (β subunit; associated protein MiRP1) (Tamargo et al., 2004; Schmitt et al., 2014). In humans, hERG expression is higher in the ventricles than the atria (Pond et al., 2000). KCNH2 and KCNE2 have been identified among the loci of mutations associated with congenital long QT syndrome (e.g., LQT2 and LQT6), which is a complex arrhythmogenic disease associated with prolonged QT interval and resultant polymorphic ventricular tachycardias, causing syncope and sudden death.

2. Existing data pertaining to allelic variation in the relevant genes that are associated with functional consequences in humans.

### Genetic variation associated with flecainide disposition

There is a lack of data for the exact mechanism for flecainide absorption across the enterocyte. Several enterocyte transporters may play a role in absorption variability. Although PEPT1 could play a role in flecainide absorption, genetic variation affecting PEPT1 expression is rarely observed considering its interaction with milk-based products (Anderle et al., 2006). MDR1—encoded by the gene *ABCB1*—protein aberrations have affected drug disposition for other cardiovascular agents (e.g., simvastatin, atorvastatin, quinidine, digoxin) (Cascorbi, 2011; Keskitalo et al., 2008), but limited data exist for flecainide (Horie et al., 2014; Hu et al., 2012). Several single nucleotide polymorphisms (SNPs) (e.g., *SLC22A2*) are associated with diminished OCT2 expression and function (Klaassen and Aleksunes, 2010). *In vitro*, *SLC22A2* variants (*SLC22A2* c.596C>T, c.602C>T, c.808 G>T) were associated with diminished metformin, a known OCT2 substrate, OCT2-mediated transport activity (Song et al., 2008). *In vivo*, available data with patient’s dosed with metformin have demonstrated diminished renal clearance in those with the *SLC22A2* c.808G>T (p.A270S) variant (Wang et al., 2008). However, there is no *in vitro* or *in vivo* data to suggest differences in OCT2-mediated flecainide transport, which remains to be explored.

The vast majority of the literature regarding the influence of genetic variation on flecainide disposition lies with cytochrome p450. Mikus et al. (1989) found the first evidence of altered flecainide disposition without renal, liver, or cardiac function abnormalities observed in poor metabolizers (PMs) of sparteine/debrisoquin, now known as CYP2D6. That study with healthy volunteers revealed a nearly 1.7-fold increase in AUC and elimination half-life and nearly 40% reduction in flecainide clearance. Not surprisingly, there was a 1.8-fold increase of unchanged flecainide in the urine in CYP2D6 PMs, consistent with inadequate flecainide alkylation to MODF, which occurs predominantly via CYP2D6 (Gross et al., 1989). As noted above, flecainide is a racemic mixture of R (−) and S (+) enantiomers, and preferential stereoselectivity has been demonstrated, with the R (−) enantiomer having greater CYP2D6 intrinsic clearance relative to the S (+) enantiomer in human liver microsomal incubations (Doki et al., 2015). Interestingly in patients with normal CYP2D6 expression (denoted as EMs), there has been no significant difference in AUC or clearance observed between the enantiomers (Gross et al., 1989; Doki et al., 2015). However, Gross et al. (1989) demonstrated in a cohort of PMs an approximate 30% increase in R (−) flecainide exposure (AUC) compared to S (+) flecainide and a subsequent 30% decrease in oral clearance, suggesting that CYP2D6 does have some stereoselective effect on flecainide. However, no difference in antiarrhythmic activity have been observed between the two enantiomers (Banitt et al., 1986). It remains unknown if S (+) enantiomers have a separate metabolism pathway and whether it contributes to inter-individual variability. More broadly, increased flecainide systemic exposure and diminished oral clearance of flecainide in PMs compared to EMs has been observed, demonstrating that CYP2D6 does impact flecainide disposition (Doki et al., 2006; Doki et al., 2009; Lim et al., 2008; Lim et al., 2010; Gross et al., 1989; Mikus et al., 1989). The CYP2D6*10 allele—phenotypically an intermediate metabolizer (IM) —does appear to significantly affect flecainide exposure. Lim et al. (2008), in a small single dose study, described a trend toward increased AUC compared to wild-type controls. In a larger and more heterogenous study, Doki et al. (2006) observed a 2- and 1.5-fold difference in the concentration to dose (C/D) ratio and clearance between IMs and homozygous EMs, respectively. However, there were no poor metabolizers included in this study. Funck-Brentano et al. (1994) and Tenneze et al. (2002) have found that CYP2D6 polymorphisms do not significantly alter flecainide’s pharmacokinetics or pharmacodynamics. However, these studies described the parameters based on phenotypic profiles as opposed to genotype, which is not reported. As previously described, CYP1A2 contributes to MODF formation; however, there are insufficient pediatric data to suggest that CYP1A2 polymorphisms contribute to alterations of flecainide’s dose–exposure–response relationship. Doki et al. (2009) suggested that elderly patients with age-related decreased expression of CYP1A2 had a 1.5-fold higher exposure to flecainide acetate than middle aged-patients. More importantly, this effect was much more dramatic in CYP2D6 PMs as CYP1A2 is an alternate pathway in CYP2D6 PMs. Overall, the CYP2D6 genotype appears to influence the pharmacokinetics of flecainide, with more data needed to ascertain this effect on pharmacodynamic endpoints, whether that be decreased arrhythmia or adverse pro-arrhythmic events.

### Genetic variation associated with flecainide response

Na_v_1.5 channels, encoded by the gene *SCN5A*, represent the majority of the pharmacodynamic effects of flecainide (Jiang et al., 2020; Abriel, 2010). Most *SCN5A* mutations result in cardiac electrophysiologic pathology, and less is known regarding the effect of *SCN5A* genetic variation on flecainide’s response. One gene variant in *SCN5A* (c.5369A>G, D1790G), located in the C-terminus location of the protein and associated with LQT3, has demonstrated that carriers of the mutation had a significant response to flecainide compared to controls (Benhorin et al., 2000). Despite concerns about sodium channel function in those with *SCN5A* c.5369A>G mutations, there is no evidence that the α-subunit properties were affected; in fact, the pathophysiologic effects of this protein aberration are due to changes in α- and β-subunit interactions (An et al., 1998). Conversely, a *SCN5A* c.5624T>C mutation (M1875T), a gene responsible for familial atrial fibrillation located in the C-terminus, results in an attenuated flecainide response compared to controls (O'Reilly et al., 2023). Another rare gain of function mutation of *SCN5A* (c.665G>A, R222Q), associated with multifocal ectopic Purkinje-related premature contractions syndrome, leads to premature action potentials during repolarization. Those with *SCN5A* c.665G>A have been successfully treated with flecainide, resulting in reduced ectopic beat burden (Ventrella et al., 2024; Calloe et al., 2018; Ter Bekke et al., 2018; Leventopoulos et al., 2021). Overall, mutations in *SCN5A* mutations are quite promising for future investigations related to the variability of flecainide’s response independent of the abovementioned flecainide disposition pathways.

Ryanodine receptor 2 proteins, encoded by the gene *RYR2*, are one of the largest ion channels found on the sarcoplasmic reticulum of cardiac myocytes and are instrumental in the regulation of cardiomyocyte excitation–contraction coupling (Steinberg et al., 2023). The gain or loss of function mutations in the *RYR2* gene can result in CPVT or calcium-release deficiency syndrome (CRDS), respectively (Steinberg et al., 2023). In CPVT, the gain of function mutations lead to increased calcium release during diastole, resulting in premature depolarizations and subsequent ventricular arrhythmia (Wleklinski et al., 2020; Liu et al., 2006). A majority of these mutations are located within the N-terminus of RyR2. There has been an absence of specific *RYR2* mutations that have resulted in alterations of flecainide response; however, like *SCN5A*, it requires more in-depth analysis to determine gene variants that might predict responders *versus* non-responders.

The I_Kr_ channel is encoded by the genes KCNH2 (α subunit; associated protein hERG) and KCNE2 (β subunit, associated protein MiRP1) (Tamargo et al., 2004; Schmitt et al., 2014). KCNH2 and KCNE2 have been identified as loci of mutations associated with LQT2 and LQT6 of the Romano–Ward variant of congenital LQTS, respectively (Schmitt et al., 2014; Roden et al., 2002; Splawski et al., 2000). More than 100 mutations in the KCNH2 gene have been described, including frameshifts, insertions, deletions, and missense and nonsense mutations (Schmitt et al., 2014; Splawski et al., 2000; Zhou et al., 1998; Rajamani et al., 2002). Mutant channels result in a net reduction in outward K+ current during repolarization that can result from different mechanisms, including the generation of nonfunctional channels, altered channel gating, and abnormal protein trafficking (Tamargo et al., 2004). Such variations may play a role in the variability observed with flecainide response and represent an area of potential research.

3. Existing data pertaining to the developmental profile (ontogeny) of the key pathways involved in flecainide disposition and response.

### Ontogenic differences in flecainide disposition

Specific patient factors (e.g., percentage of fat stores, systemic protein concentration) can be developmental in nature and thereby influence drug disposition differently for developing children relative to fully matured adults (Kearns et al., 2003; Wagner and Abdel-Rahman, 2013). Flecainide’s moderate lipophilicity is highly relevant for developing children, where drug distribution could potentially be more discordant than adults due to alterations in the percentage of weight for water and fat stores in infants. For example, ∼80% of a newborn’s weight is composed of water, with many limitations in fat stores (Kearns et al., 2003). These steadily increase during infancy; the adult percentage of weight comprised of fat is achieved at approximately 36 months (Wagner and Abdel-Rahman, 2013; Funk et al., 2012). Thus, infants with a smaller percentage of fat stores could theoretically have less passive diffusion into their peripheral tissues and higher systemic exposure after a dose of a lipophilic agent (i.e., flecainide). The volume of distribution (Vd) in adults after oral dosing is very large (∼512L), suggesting that the agent has a high degree of lipophilicity (Holmes and Heel, 1985), although its modest protein binding suggests otherwise. As noted above, neonates and young infants have diminished fat stores compared to adults (Wagner and Abdel-Rahman, 2013), and it could theoretically be expected that flecainide, with its modest lipophilic profile, would demonstrate lower Vd in infants and young children. However, this is largely not observed in other lipophilic drugs, as they tend to freely associate with cellular components in tissues (e.g., heart) other than adipose. For example, some drugs with large Vds accumulate in tissues through their affinity for acidic subcellular compartments (e.g., lysosomes), which are enriched in the liver, lung, heart, brain, and kidneys (MacIntyre and Cutler, 1988; Yokogawa et al., 2002). This developmental change in tissue distribution, especially in the heart, could be another source of variability in the dose–exposure–response relationship in the developing infant. The degree of distribution to peripheral tissues, including adipose tissue, for lipophilic drugs is controversial (Kyler et al., 2019). Overall, there are few concrete data to confirm higher systemic exposure amongst infants compared to adults due to limitations in fat stores, but this must be explored in the future for those dosed with flecainide.

It is not known if flecainide’s protein binding is altered in neonates and further varied in neonatal cardiovascular disease states. Quantitatively, it known that that albumin and α1-acid glycoprotein concentrations are lower in neonates and could result in higher unbound flecainide than adults (Koukouritaki et al., 2004). Additionally, the reduced binding affinity of fetal albumin and the presence of endogenous or exogenous substances that compete for protein binding sites, including free fatty acids, bilirubin, and other drugs, have been described for other drug agents (Windorfer et al., 1974; Nau et al., 1984; Rane et al., 1971). Notwithstanding, this impact of protein binding on the distribution of flecainide in the developing child, especially neonates, requires further study.

As summarized in Table 1, MDR1, OCT2, and PEPT1 transporters and CYP1A2 and CYP2D6 have become the best proteins (genes) to evaluate for precision-based flecainide therapy for pediatrics. Admittedly, there are still some knowledge gaps in regard to transporter ontogeny. Peptide transporters in chicks increase expression from the late embryonic stages to birth, where such expression peaks (Zwarycz and Wong, 2013). The trajectory of peptide transport expression for infants and small children remains unknown and requires further elucidation. Within the last decade, the proteomic evaluation of pediatric and adult hepatic tissue has demonstrated some ontogenic changes in influx and efflux drug transporters (Prasad et al., 2016). Of the proteins evaluated, OCT1, OATP1B3, and MDR1 had significant increases in protein expression from the neonatal period to adulthood, with MDR1 demonstrating significant changes at each age group. Notably, OCT2 was not evaluated in this cohort but should be evaluated in future iterations.

The ontogeny of CYPs in humans occurs in distinct patterns that are isoenzyme-specific (Hines, 2008). CYP2D6 is a member of a gene locus that contains no other significant members, but it alone is responsible for the metabolism of over 10% of clinically relevant drugs (Williams et al., 2004). The ontogeny of CYP2D6 is characteristic of a Group 3 pattern of expression proposed by Hines (2008). This translates to functional activity that is detectable but low at birth (∼5% of that observed in adults), increasing to nearly 70% of that seen in adults at 5 years of age (Treluyer et al., 1991); a majority of that maturation occurs in the first few months (Treluyer et al., 1991; Blake et al., 2007; Stevens et al., 2008). However, the *in vivo* functionality of *CYP2D6* in genetic variants did not change as a function of age, suggesting concordance between the phenotype and genotype within the first month of life (Blake et al., 2007). For a neonate with SVT, this could be a potential source of variability and contribute to the toxicity experienced in some neonates compared to older infants (Palmen et al., 2023).

CYP1A2 ontogeny is relevant to flecainide disposition, especially in CYP2D6 PMs (Doki et al., 2009), as described above. CYP1A2 expression decreases in late adulthood and has a functional effect on flecainide exposure and disposition (Doki et al., 2009). This exact mechanism could also negatively affect our neonatal population where CYP1A2 expression is low. *In vitro and in vivo*, CYP1A2 is not detected in fetal liver samples (Hakkola et al., 1994; Bieche et al., 2007; Lee et al., 1991; Yang et al., 1995; Cazeneuve et al., 1994; Sonnier and Cresteil, 1998). Even after parturition, CYP1A2 ontogeny is delayed, with expression ten-fold less than in adults (Cazeneuve et al., 1994; Carrier et al., 1988). Even in young infants (1–3 months) and young children (1–9 years), expression of CYP1A2 is only 10%–15% and 50%–55% of adult levels, respectively (Sonnier and Cresteil, 1998). However, CYP1A2 maturation can be accelerated in formula-fed infants compared to breastfed (Blake et al., 2006; Le Guennec and Billon, 1987) and also should be considered in developing children who are prescribed flecainide.

### Ontogenic differences in flecainide response

Overall, little is known about the ontogeny of the proteins related to flecainide response. In murine models, Na_v_1.5 channels are expressed in fetal life with ∼30% expression relative to adult levels. Expression increases *postpartum* within the first 2 weeks of age and approaches ∼60% expression relative to adult levels during adolescence (Haufe et al., 2005). The ontogenesis of Na_v_1.5 channels in human cardiac myocytes remains unknown. Amphipod embryologic and development studies support RyR2 upregulation throughout the developmental process, although more so during the middle and late stages (McAndry et al., 2022). RyR2 is critical for the development of cardiac functioning and continues to play a vital role in excitation–contraction coupling in the heart throughout human life, exemplified the presence of mutations/genetic variance and arrythmia development (McAndry et al., 2022). Although attention has been paid to structural components and expression patterns of the I_Kr_ channel, as described above, less is known about the developmental pattern or ontogeny of the I_Kr_ channel in humans. This represents an area of future research to better understand the differences seen in flecainide response.

## Summary

Flecainide is a highly effective drug therapy for the prevention of lethal arrhythmia in the pediatric population. However, its narrow therapeutic index makes drug dosing decisions challenging for clinicians. There are no current Clinical Pharmacogenetics Implementation Consortium (CPIC) guidelines to assist with flecainide dosing for both the adult and pediatric populations. There are Dutch Pharmacogenomic Working Group (DPWG) guidelines for flecainide dosing based on the CYP2D6 genotype. Those with IM (e.g., *CYP2D6* *1/*7) and PM (*CYP2D6* *3/*3) phenotypes are recommended to start dosing at 75% and 50% of the standard dose, respectively, with simultaneous pharmacokinetic (e.g., plasma flecainide level) and pharmacodynamic (e.g., ECG QRS/QT duration) assessments at baseline and during treatment (PharmGKB API, 2024). Although stereoselective flecainide CYP2D6-mediated metabolism was not demonstrated in those with normal CYP2D6 function, despite R (+) flecainide’s enhanced affinity to CYP2D6, it was determined that genotypes associated with diminished CYP2D6 function had diminished R (+) flecainide metabolism. Animal models suggest no pharmacodynamic differences between the enantiomers. There are no human data to validate this observation, and this requires investigation. Although the *CYP2D6* genotype appears to represent the majority of inter-individual variability, several other co-variates have been outlined within this review as opportunities for further investigation. For example, drug transporters could play a complimentary role to *CYP2D6* and, by extension, the *CYP1A2* genotype that is responsible for the variability in flecainide’s systemic exposure. Priority should be given to the influence of MDR1 and OCT2 on flecainide’s systemic exposure and the consequences of the gene variation of these transporters. For neonates with incessant tachyarrhythmia and who are refractory to first-line treatment (e.g., beta blockers), the challenges for precision dosing are more complex given the age-related changes to protein binding and CYP ontogeny. Collectively, these ontogenic factors must be evaluated to determine the role of development on flecainide systemic exposure variability. Future research must collect those *in vitro* and *in vivo* data to develop and validate mechanistic pharmacologic models (e.g., popPK, PBPK) that will take into consideration multiple co-variates that can be used to ultimately develop individualized dosing tools for pediatric and maternal-fetal patients dosed with flecainide.

## Abbreviations

AUC, area under the curve; CRDS, calcium release deficiency syndrome; CAST, Cardiac Arrhythmia Suppression Trial; CHD, congenital heart disease; I_Kr_, delayed rectifier K+ channel; FDA, Food and Drug Administration; LQT, long QT syndrome; Cmax, maximal concentration; MODF, m-O-dealkylated flecainide; MODLF, m-O-dealkylated lactam flecainide; MDR1, multidrug resistance protein 1; OCT, organic cation transporter; PEPT1, peptide transporter 1; P-gp, P-glycoprotein; RyR2, ryanodine receptor 2; SVT, supraventricular tachycardia.
